# Revisiting Fat Content in Bone Lesions: Paradigms in Bone Lesion Detection

**DOI:** 10.3390/diseases13070197

**Published:** 2025-06-27

**Authors:** Ali Shah, Neel R. Raja, Hasaam Uldin, Sonal Saran, Rajesh Botchu

**Affiliations:** 1 Department of Radiology, Nottingham University Hospitals NHS Trust, Nottingham NG7 2UH, UK; ali.shah1@nhs.net; 2 Department of Radiology, Leicester Royal Infirmary, Infirmary Square, Leicester LE1 5WW, UK; neel.raja@nhs.net; 3 Department of Musculoskeletal Radiology, Royal Orthopaedic Hospital, Bristol Road South, Northfield, Birmingham B31 2AP, UK; hasaam.uldin1@nhs.net; 4 Department of Radiology, All India Institute of Medical Sciences (AIIMS), Rishikesh 249203, India; sonalsaranmalik@gmail.com

**Keywords:** intraosseous, bone lesion, intraosseous fat, chemical shift imaging

## Abstract

Bone lesions encountered as part of radiology practice can bring diagnostic challenges, both when encountered incidentally or suspected as a primary bone lesion, and in patients at risk of metastases or marrow-based malignancies. Differentiating benign from malignant bone marrow lesions is critical, yet can be challenging due to overlapping imaging characteristics. One key imaging feature that can assist with diagnosis is the presence of fat within the lesion. Fat can be present either macroscopically (i.e., visible on radiographs, computed tomography (CT), and conventional magnetic resonance imaging (MRI)), or microscopically, detected through specialised MRI techniques such as chemical shift imaging (CSI). This comprehensive review explores the diagnostic significance of both macroscopic and microscopic fat in bone lesions and discusses how its presence can point towards benignity. We illustrate the spectrum of fat-containing bone lesions, encompassing both typical and atypical presentations, and provide practical imaging strategies to increase diagnostic accuracy by utilising radiographs, CT, and MRI in characterising these lesions. Specifically, CSI is highlighted as a non-invasive method for evaluating intralesional fat content, to distinguish benign marrow entities from malignant marrow-replacing conditions based on quantifiable signal drop-off. Furthermore, we detail imaging pitfalls with a focus on conditions that can mimic malignancy (such as aggressive haemangiomas) and collision lesions. Through a detailed discussion and illustrative examples, we aim to guide radiologists and clinicians in recognising reassuring imaging features while also identifying scenarios where further investigation may be warranted.

## 1. Introduction

Bone lesions are commonly encountered during image reading as part of the clinical practice of any radiologist. Equally, they can be a source of significant diagnostic uncertainty, particularly in individuals with a known history of malignancy. Accurate lesion characterisation is paramount to guide appropriate management and avoid unnecessary biopsy or overtreatment. While many bone lesions can be confidently diagnosed based on classic imaging features, others may present with ambiguous characteristics.

A central diagnostic clue in the evaluation of bone lesions is the presence of fat, either macroscopic and therefore visible on standard imaging techniques such as radiographs, computed tomography (CT), and magnetic resonance imaging (MRI) [1], or microscopic, which requires specialised MRI techniques such as chemical shift imaging (CSI) for detection. Recognising fat content within bone lesions provides useful diagnostic information that can help differentiate benign entities, such as lipomas or typical haemangiomas, from sinister lesions such as malignancy and metastases.

This review aims to provide a straightforward, detailed, and practical guide to recognise classic and atypical fat-containing bone lesions, understanding the diagnostic relevance of intralesional macroscopic and/or microscopic fat, applying CSI in clinical practice, and knowing when fat is a reassuring feature versus when it demands further work-up.

## 2. Typical and Atypical Fat-Containing Bone Lesions

Fat-containing bone lesions span a spectrum from benign to potentially malignant conditions. Recognising these lesions relies on identifying fat within the lesion and integrating this finding with the clinical context and other imaging features.

### 2.1. Typical Fat-Containing Lesions

Several benign lesions commonly contain macroscopic fat, visible across multiple imaging modalities:

- Intraosseous lipoma;
- Haemangioma;
- Other entities, e.g., bone infarct.

#### 2.1.1. Intraosseous Lipoma

Intraosseous lipoma is the most common lipogenic bone lesion, although rarely encountered in clinical practice. Given its overall appearances can mimic certain other conditions and because the presence of intralesional fat is often detected on plain radiographs, further characterisation with cross-sectional imaging is often not performed. This may result in the underreporting of this lesion, which perhaps occurs more than its quoted prevalence of less than 1 per 100 cases [2].

It typically affects adults in the 4th to 5th decades, with a slight male predominance. While often asymptomatic and discovered incidentally, up to 66% can present with localised pain. Rarely, lesions lead to pathological fractures or palpable masses.

Frequent locations include the intertrochanteric region of the femur (34%), calcaneus (8%), tibia (13%), fibula (10%), ilium (8%), humerus (5%), and ribs (5%) [2]. Most lesions are metaphyseal (when in long bones), intramedullary, and solitary, though rarely they can be intracortical and also present as multiple lesions (intraosseous lipomatosis). Diaphyseal location is also not uncommon.

##### Pathology and Histology

Perhaps not a true tumour, the lesion is thought to reflect a hamartomatous overgrowth of fat during red-to-yellow marrow transition, particularly in bones with sparse trabeculae and more fat, such as the femur and calcaneus. Some cases show ischaemic features, suggesting evolving fat necrosis and secondary calcification. Milgram [3], a key figure in studying intraosseous lipomas, described three stages of the lesion’s life cycle:

- Stage 1: Pure fat without any degeneration.
- Stage 2: Fat with some necrosis and calcification.
- Stage 3: Advanced changes like fat necrosis, cyst formation, calcification, and new bone growth.

##### Imaging Features

The overall appearances depend on the quantity of intralesional fat, with the lesion containing various amounts of fat, bone, fibrous tissue, and cystic degeneration.

On radiographs in early stages, the lesion appears as a well-defined, radiolucent (darker) area and can cause mild bone expansion, especially in thinner bones like the fibula. It resembles other benign lesions like bone cysts or fibrous dysplasia. CT can be useful in confirming the fat content (seen as low-density areas) and can help distinguish lipomas from surrounding marrow if a capsule is present.

**Figure 1:**
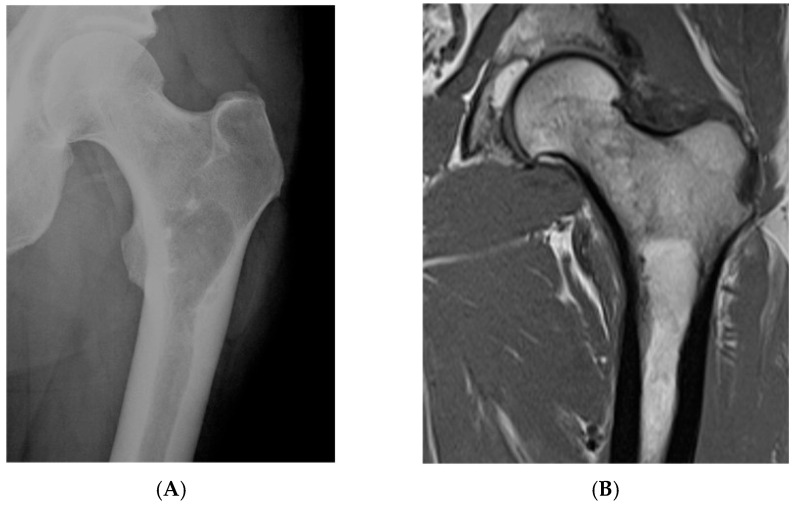

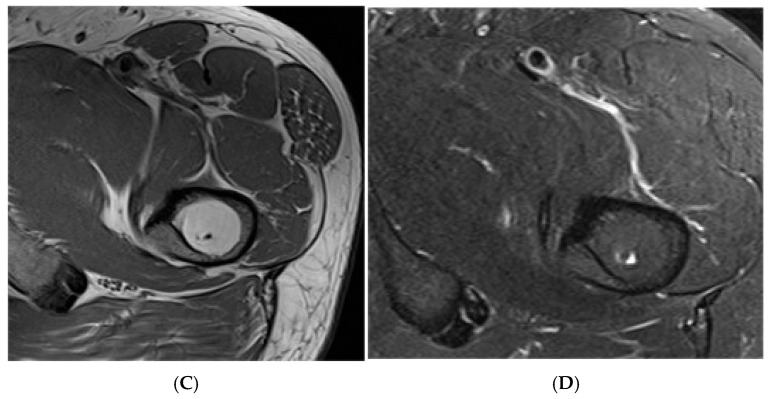
Intraosseous lipoma, left proximal femur. (**A**) AP radiograph proximal femur depicting well-defined, lucent bone lesion. (**B**) Coronal T1W MRI depicting fat-containing lesion. (**C**,**D**) Axial MRI T1W and STIR (respectively) demonstrating fat content without and with fat suppression.

**Figure 2:**
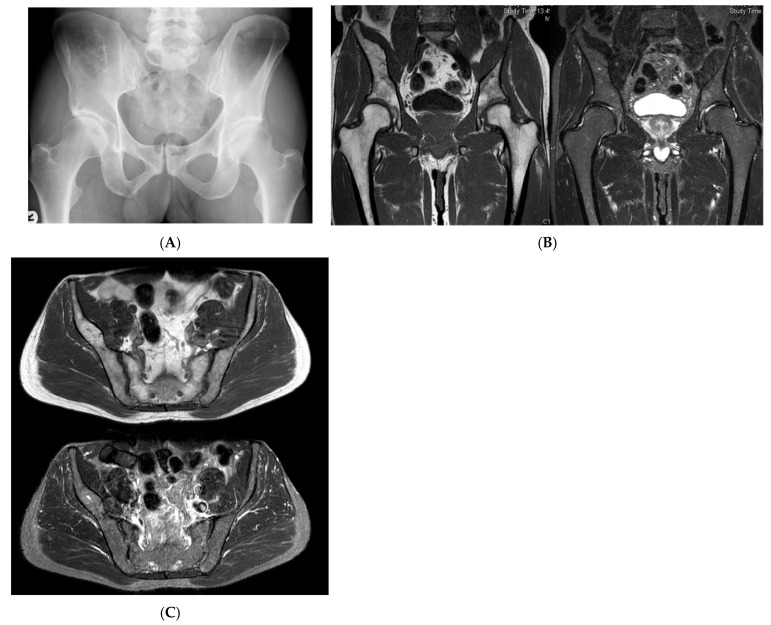
Intraosseous lipoma, right iliac bone. (**A**) AP radiograph demonstrating a right iliac bone lesion. (**B**) Coronal TW1 and STIR MRI side by side, demonstrating a right iliac fat-containing bone lesion exhibiting fat suppression. (**C**) Axial T1W MRI showing a right iliac bone lesion and axial STIR MRI of the same lesion with fat suppression.

**Figure 3:**
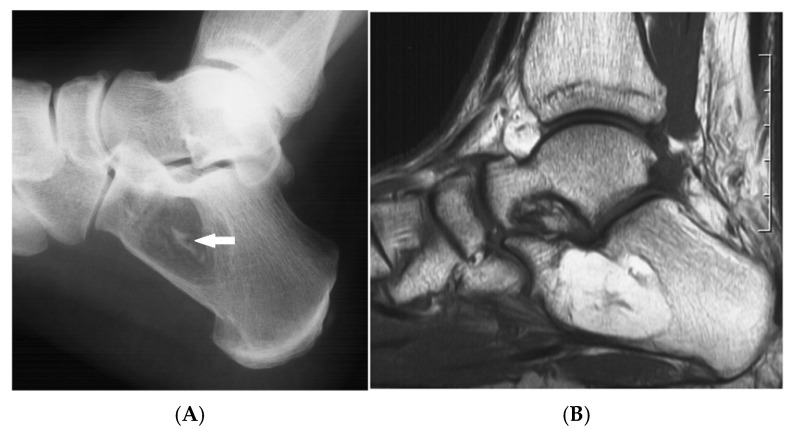
Intraosseous lipoma, calcaneum. (**A**) Plain radiograph demonstrating the classic location of the lesion with “bull’s eye” sign (solid white arrow). (**B**) Sagittal T1W MRI with fat-containing lesion.

MRI generally demonstrates the lesion as hyperintense on T1 and T2, essentially following the fat signal, and hypointense on fat-suppressed sequences. In some cases, the fat content may look very similar to surrounding marrow, making it hard to distinguish, and the presence of a capsule or the expansion of bone can be helpful clues [2] (Figure 1, Figure 2 and Figure 3).

Advanced stages of the lesion may show central or peripheral calcification, described as a distinctive “bull’s-eye” appearance, or ossification, occasionally mimicking enostosis, chondroid lesions, or osteonecrosis. Asymptomatic lesions require no treatment, whilst symptomatic cases may be treated with curettage and bone grafting. Recurrence and malignant transformation are rare.

#### 2.1.2. Haemangioma

These constitute less than 10% of all primary bone neoplasms and only 0.5% to 1% of all intraosseous tumours [4,5]. They predominantly affect the vertebral column and skull (75%), with occasional occurrences in facial bones such as the zygoma and mandible, as well as long bones [6]. These lesions are often asymptomatic and discovered incidentally during imaging for unrelated conditions, with 25% of cases presenting in the 4th-5th decade [4]. Specifically, vertebral haemangiomas constitute 28% of all skeletal haemangiomas, with most lesions being at the thoracic spine [7]. They can either involve a part of or the whole of the vertebral body, including posterior elements, and can be multiple in up to 25–30% cases [8].

Intraosseous haemangiomas located outside the mobile spine and calvarium tend to exhibit more aggressive imaging characteristics than their vertebral counterparts. These may include cortical permeation, associated soft tissue masses, and ill-defined areas of increased density resembling osteoid matrix [9]. In facial bones, cosmetic deformity may be a concern, and lesions in the orbit can lead to proptosis or visual disturbances [5].

##### Pathology and Histology

Intraosseous haemangiomas are slow-growing tumours in the medullary cavity (55%), characterised by proliferating endothelial cells that form vascular spaces of varying sizes, supported by a fibrous connective tissue stroma. They can also be periosteal (33%) and intracortical (12%). Bony trabeculae may be intermixed within the lesion. Osseous haemangiomas can be divided according to the predominant type of vascular channel into capillary (small vessels), cavernous, arteriovenous, or venous [7].

##### Imaging Features

On radiographs, they are seen as a radiolucent lesion demonstrating a coarse, vertical, trabecular pattern, with osseous reinforcement (trabecular thickening) adjacent to the vascular channels that have caused the bone resorption. This pattern has been likened to a stress response and called the “honeycomb” or “corduroy” pattern [7].

Being a common incidental finding on CT, they are usually not a cause for concern [8]. CT provides a detailed assessment of bony architecture, often showing a well-defined lytic lesion with internal trabeculations interspaced within a fat attenuation matrix. It is because of these trabeculations and their structural support that vertebral fractures are less common due to the reinforcement [7]. When seen in cross-section, the trabeculations appear as tiny punctate areas described as the “polka-dot” sign.

On MRI, lesions typically exhibit high signal intensity on T2-weighted images due to their vascular nature and may enhance with contrast administration. The fat content can vary, which is responsible for the range of signal intensities demonstrated on T1-weighted images, reflecting the proportion of fat vs. vascular components.

Therefore, haemangiomas on MRI are divided into three types [10]:

1. Typical: Fatty component predominates. High T1, high T2, and mildly hyperintense on STIR.
2. Atypical: Vascular component predominates. Iso- to hypointense on T1, high on T2 and STIR. Trabeculations may be less visible.
3. Aggressive: Variable fat and vascular stroma, and therefore variable T1, T2, and STIR intensities.

Aggressive haemangiomas can cause cortical destruction and expansion, mimicking malignancy. They exhibit a constellation of imaging features which may include the involvement of the neural arch and the complete replacement of the vertebral body, often accompanied by cortical expansion. A predilection for the thoracic spine has been observed, and lesions may demonstrate an irregular honeycomb or trabecular pattern, deviating from the typical vertical striations of benign haemangiomas. The presence of an associated soft tissue mass further raises concern for aggressive behaviour. Despite these findings, a sharp lesion margin with preserved adjacent normal marrow can be reassuring. Additionally, prominent or enlarged paraspinal vessels may be seen due to the lesion’s vascular nature, aiding in differentiation from non-vascular neoplasms.

**Figure 4:**
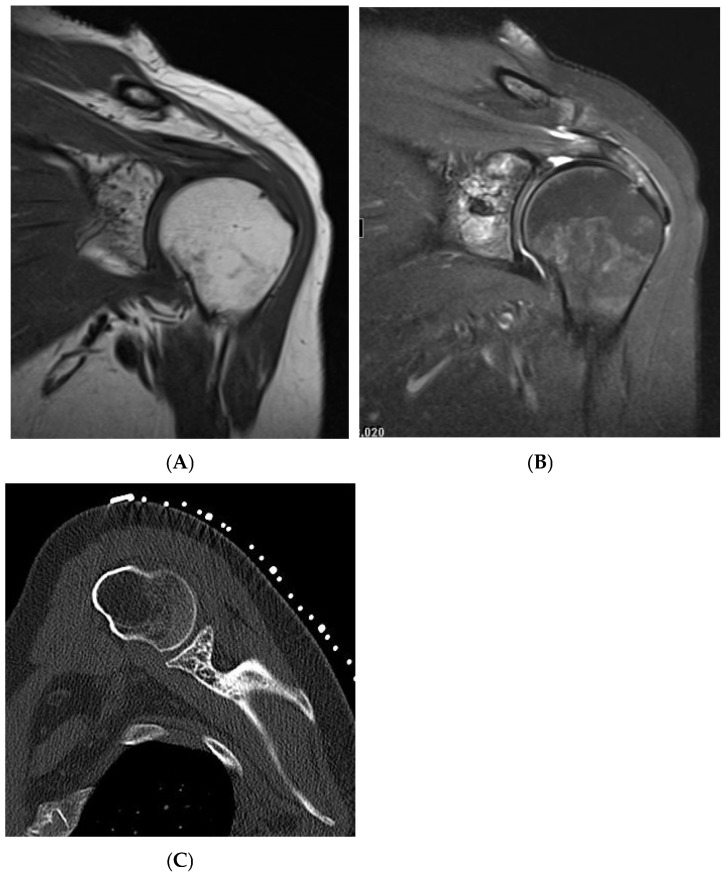
Haemangioma, left proximal humerus. (**A**) Coronal T1W MRI demonstrating a left glenoid predominantly hyperintense lesion with speckled low-intensity areas representing mineralization. (**B**) Coronal STIR MRI. (**C**) Axial CT of the left glenoid demonstrating “polka-dot” sign—low-density fat lesion with interspersed foci of mineralization.

**Figure 5:**
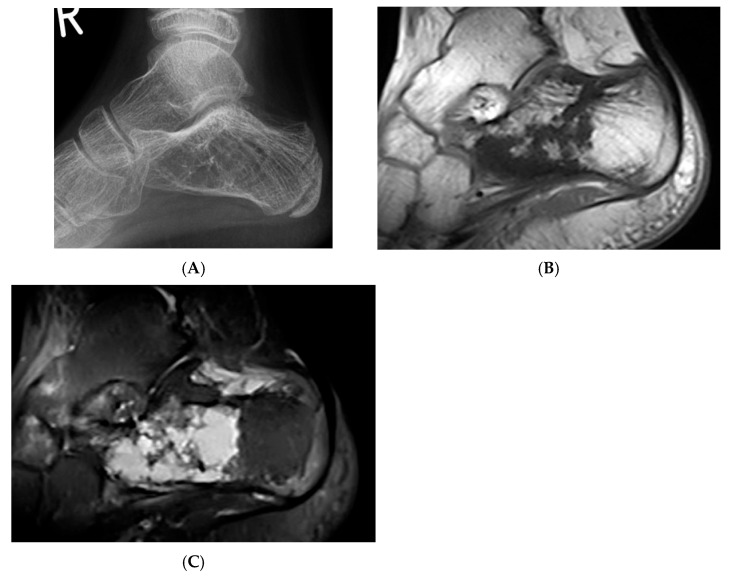
Haemangioma, calcaneum. (**A**) Plain radiograph demonstrating honeycomb pattern. (**B**) Sagittal T1W MRI and (**C**) sagittal STIR MRI demonstrating intralesional fat component (low T1 and high STIR) and septations.

**Figure 6:**
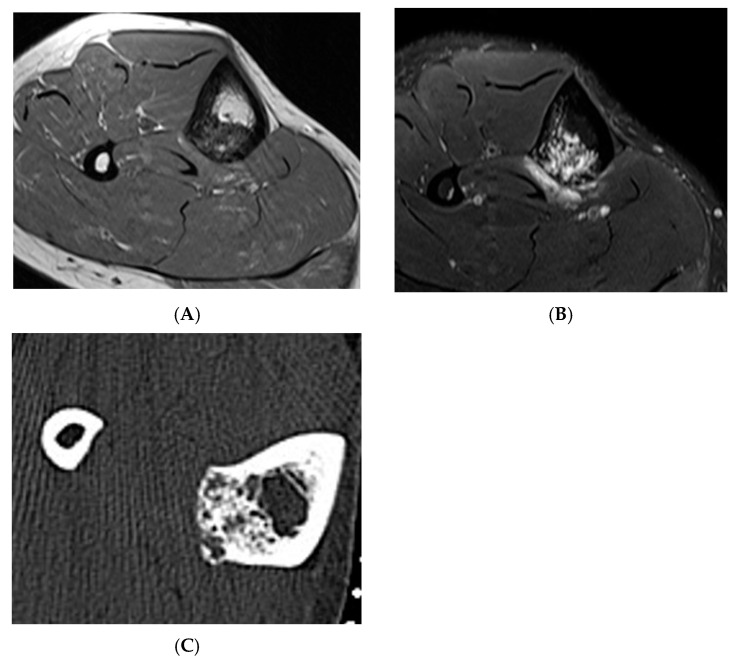
Haemangioma, tibia. (**A**) Axial T1W MRI. (**B**) Axial STIR MRI. (**C**) Axial CT demonstrating “honeycomb” pattern of intraosseous haemangioma. Low signal intensity on T1 with corresponding high SIR signal on STIR suggests the low fat content of the lesion as seen on CT with few interspersed areas of hypodensity in the lesion.

**Figure 7:**
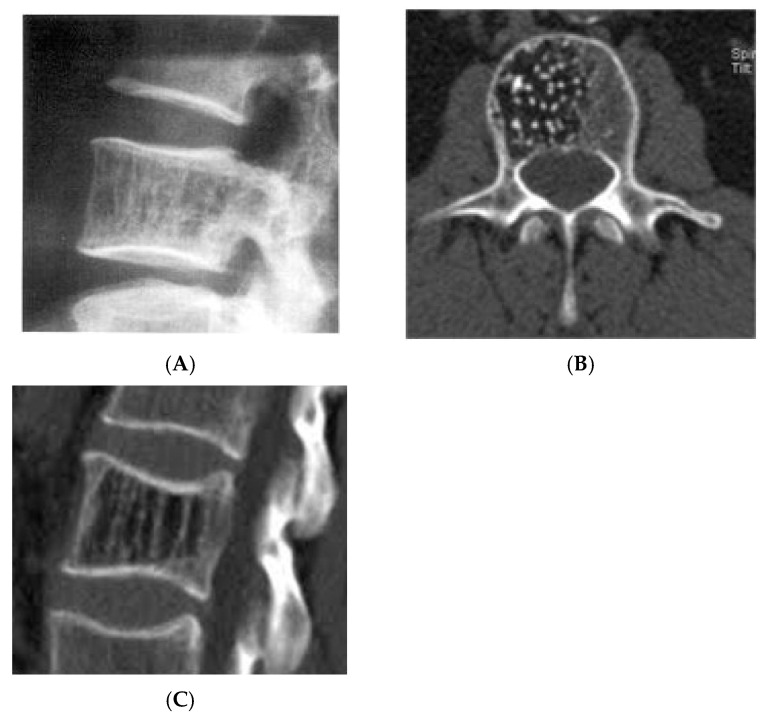
Vertebral haemangioma. (**A**) Plain radiograph lateral view demonstrating “corduroy” pattern. (**B**) Axial CT demonstrating “polka-dot” pattern. (**C**) Sagittal CT demonstrating “corduroy” pattern.

**Figure 8:**
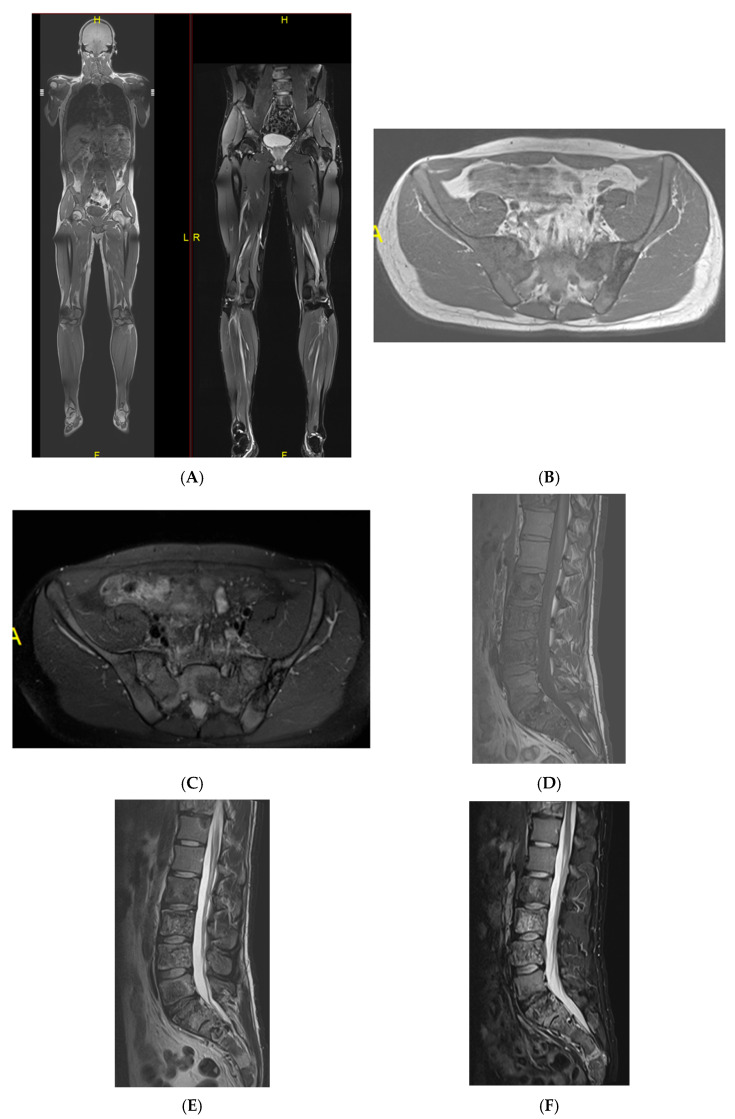

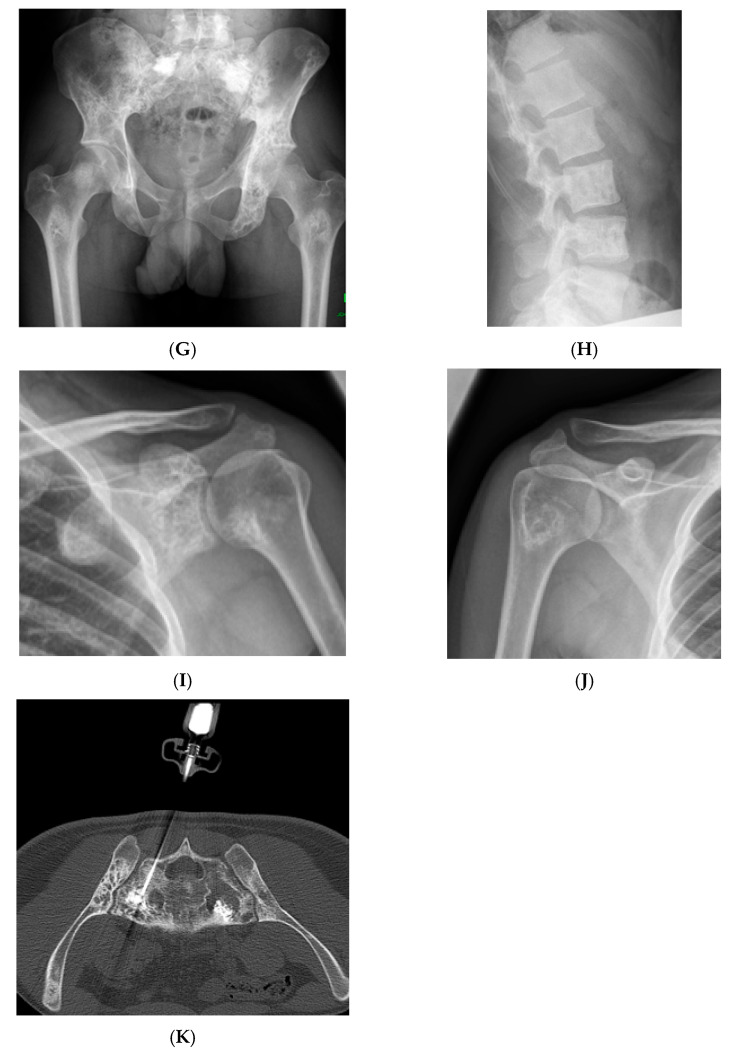
Multiple haemangiomas. (**A**) Coronal T1W MRI whole body with side-by-side coronal STIR MRI whole body showing multiple haemangiomas at the vertebral column, pelvis, and proximal femora. (**B**) Axial T1W Pelvis MRI and (**C**) STIR Pelvis MRI, further detailing the pelvis lesions. (**D**) Sagittal T1W Spine MRI with spine haemangiomas. (**E**) Sagittal T2W Spine MRI with spine haemangiomas. (**F**) Sagittal STIR Spine MRI with spine haemangiomas. (**G**) Plain radiograph, pelvis. (**H**) Plain radiograph, lumbar spine. (**I**) Plain radiograph, left shoulder. (**J**) Plain radiograph, right shoulder. All radiographs showcase the corresponding appearances of the lesion on plain films. (**K**) Axial CT pelvis with biopsy-proven haemangioma at the right hemi-pelvis.

The treatment of intraosseous haemangiomas is guided by symptoms and imaging features. Asymptomatic, non-aggressive lesions may be monitored, while symptomatic or suspicious lesions may require en-bloc resection, often preceded by embolisation to minimise intraoperative bleeding. Other treatment modalities include sclerotherapy, vertebroplasty, cryotherapy, and radiotherapy for large lesions [11] (Figure 4, Figure 5, Figure 6, Figure 7 and Figure 8).

#### 2.1.3. Other Lesions

**Figure 9:**
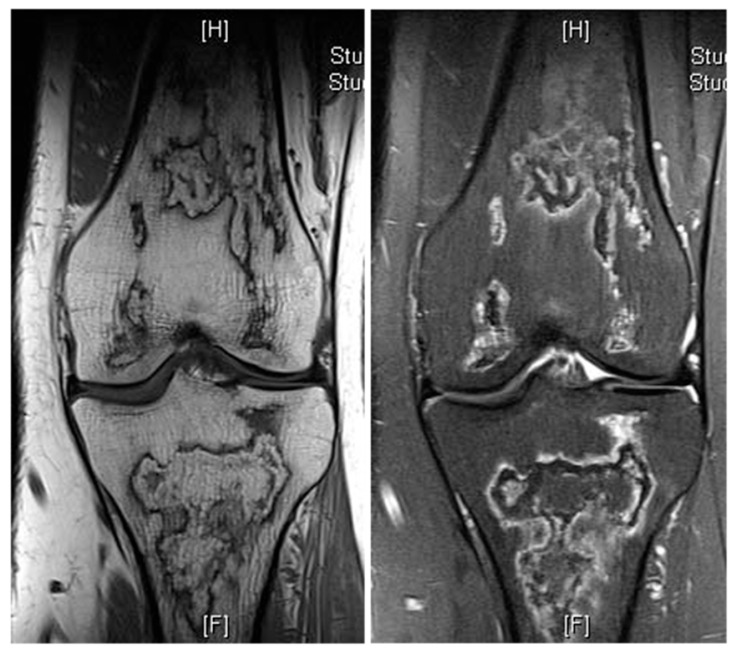
Bone infarct knee. Coronal T1W and STIR knee MRI demonstrating serpiginous, peripheral low signal intensity with central fat intensity signal (high T1 and low STIR).

Other commonly seen fat-containing benign entities include bone infarcts, a specific phenomenon of osteonecrosis in the medullary cavity of the metaphyses or diaphyses of long bones. These are usually identified incidentally and typically present on imaging as a well-circumscribed serpiginous lesion, demonstrating an outer shell-like sclerotic margin with areas of mottled bone lucency and subtle reactive sclerosis. Key imaging features include a central fatty change of the lesion. On MRI, a characteristic “double-line sign” is evident on T2-weighted images, accompanied by a geographic, map-like rim of low signal intensity encasing a central zone with fat or fluid signal characteristics [12] (Figure 9). Malignant transformations of bone infracts are rare. A review of sequential imaging where available with a progressive loss of normal fat should raise the suspicion of malignancy within bone infracts. A study has described more than 10 cases of secondary sarcomas arising from bone infarcts with majority being osteosarcomas [13].

### 2.2. Atypical Presentations and Diagnostic Challenges

**Figure 10:**
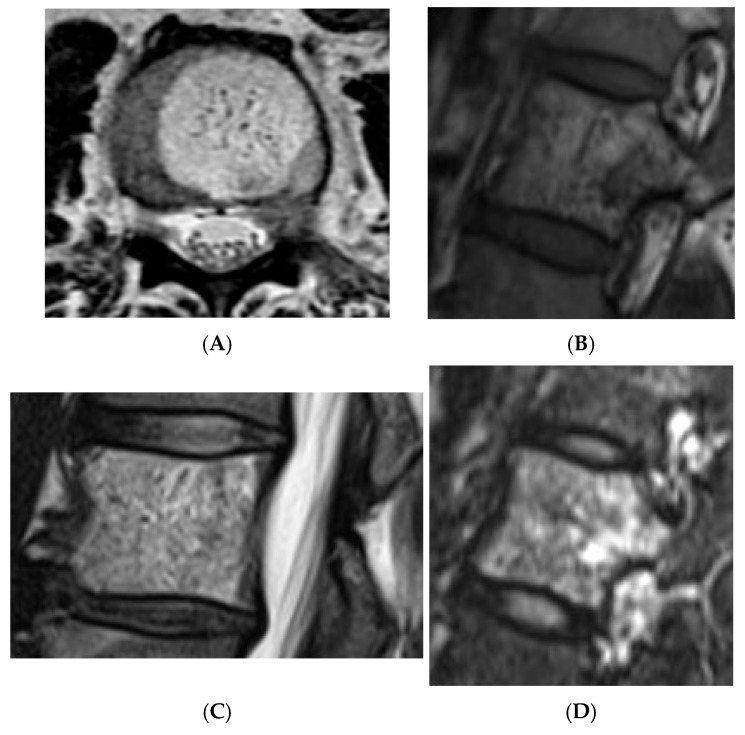
Typical haemangioma of the vertebral body. (**A**) T1W axial MRI. (**B**) T1W sagittal MRI. (**C**) T2W sagittal MRI. (**D**) STIR sagittal MRI.

**Figure 11:**
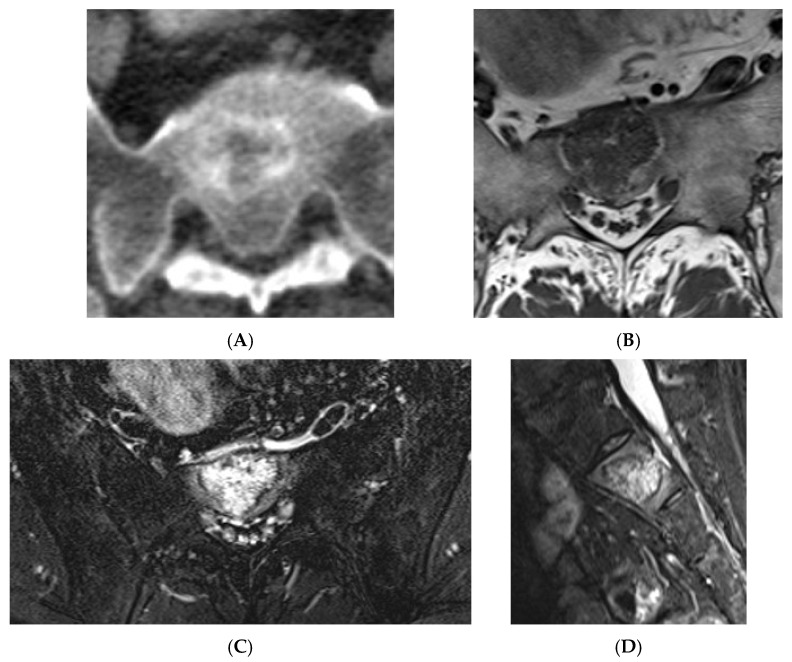
Atypical haemangioma at sacrum. (**A**) Axial CT. (**B**) T1W axial MRI. (**C**) Axial STIR MRI. (**D**) Sagittal STIR MRI.

**Figure 12:**
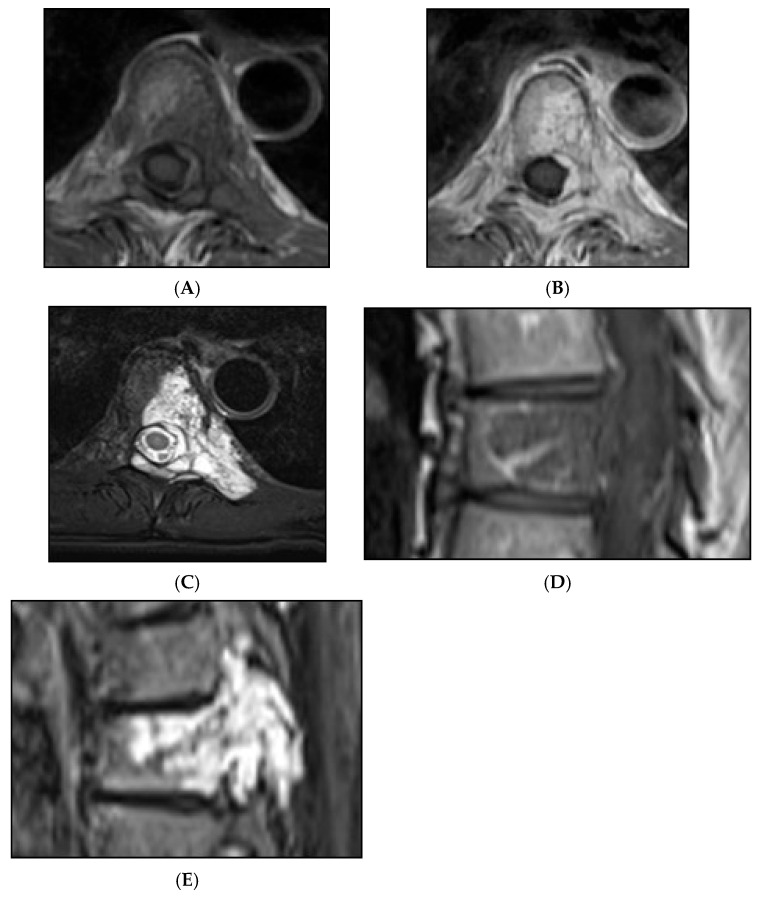
Aggressive spine haemangioma with involvement of the vertebral body and posterior elements with cortical expansion. (**A**) Axial T1W MRI. (**B**) Axial T1W post-contrast MRI. (**C**) Axial STIR MRI. (**D**) Sagittal T1W MRI. (**E**) Sagittal STIR MRI.

**Figure 13:**
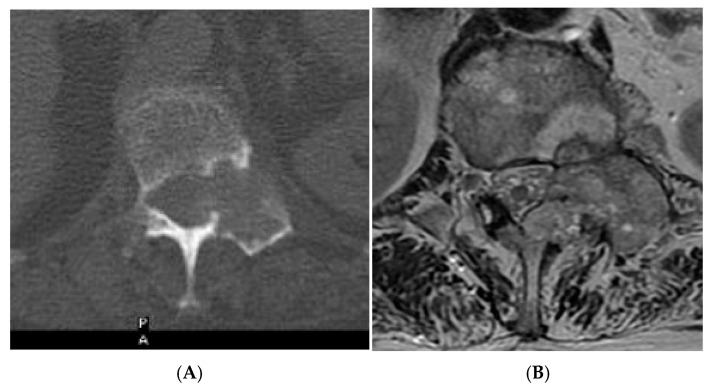

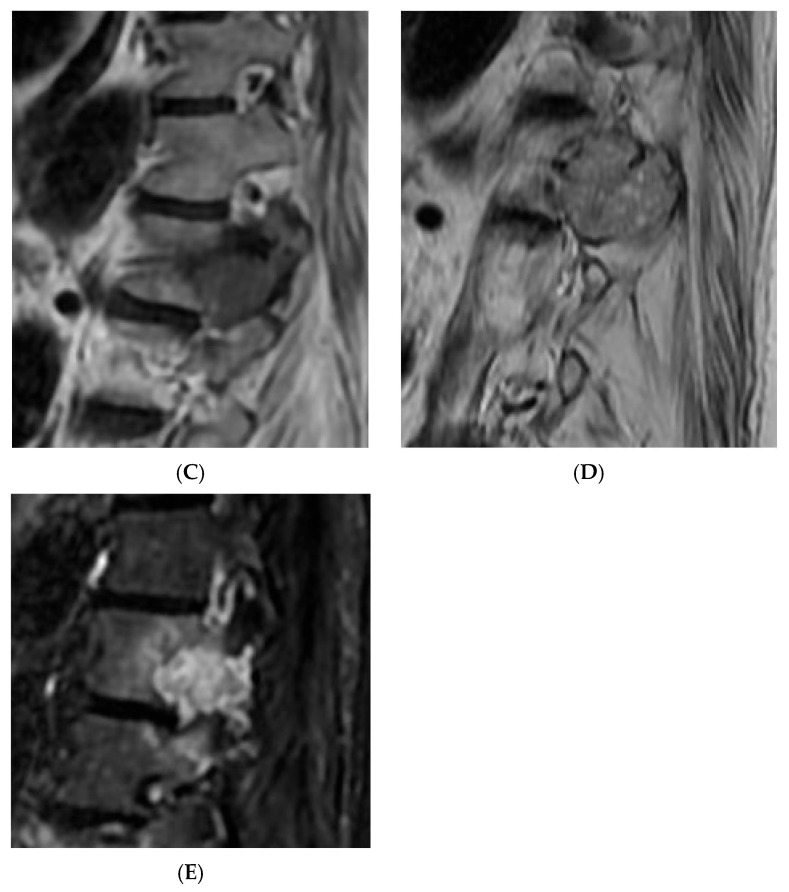
Aggressive spine haemangioma. (**A**) Axial CT showing lytic lesion within the left-side posterior aspects of the vertebral body with cortical destruction. (**B**) Axial T2W MRI with the expansile lesion encroaching within the spinal canal and neural foramen. (**C**) Sagittal T1W MRI. (**D**) Sagittal T2W MRI and (**E**) sagittal STIR MRI, demonstrating a more vascular component of the lesion along with expansile component effacing the neural foramen.

Some fat-containing lesions present atypically, as described above in the example of atypical and aggressive haemangiomas. Despite their appearance, the key differentiating feature between different types of intraosseous haemangiomas is their histologically; vertebral haemangiomas are considered hamartomatous lesions, composed of variable proportions of vascular channels within a fatty stroma [14,15]. Their appearance on MRI is thus largely determined by the ratio of these two components [15]. Typical haemangiomas are characterised by a predominance of fatty stroma, resulting in high signal intensity on T1-weighted images and signal suppression on fat-saturated sequences. In contrast, atypical haemangiomas exhibit a higher proportion of vascular channels, often accompanied by interstitial oedema and reduced fat content. These features contribute to their more heterogeneous isointense or even low signal appearance on T1-weighted imaging [16], which can mimic more aggressive or malignant pathology (Figure 10, Figure 11, Figure 12 and Figure 13). Intralesional fat content assessment and quantification can therefore assist with diagnosis and management decision-making in terms of imaging follow-up versus histological diagnosis (biopsy).

Certain infiltrative marrow disorders, such as lymphoma or leukaemia, may retain a degree of intralesional fat, and some vascular lesions may demonstrate T1 hyperintensity relative to adjacent musculature. However, these entities are rarely solitary or incidentally discovered, distinguishing them from typical benign fat-containing lesions [17].

Another important pitfall within the spectrum are collision lesions. Collision lesions are rare entities in which two histologically distinct tumours, either two primary neoplasms or a metastatic lesion infiltrating another tumour, coexist within the same anatomical site, a phenomenon reported in various organs including the liver, the adrenal gland, the genitourinary tract, and the brain [18,19,20].

**Figure 14:**
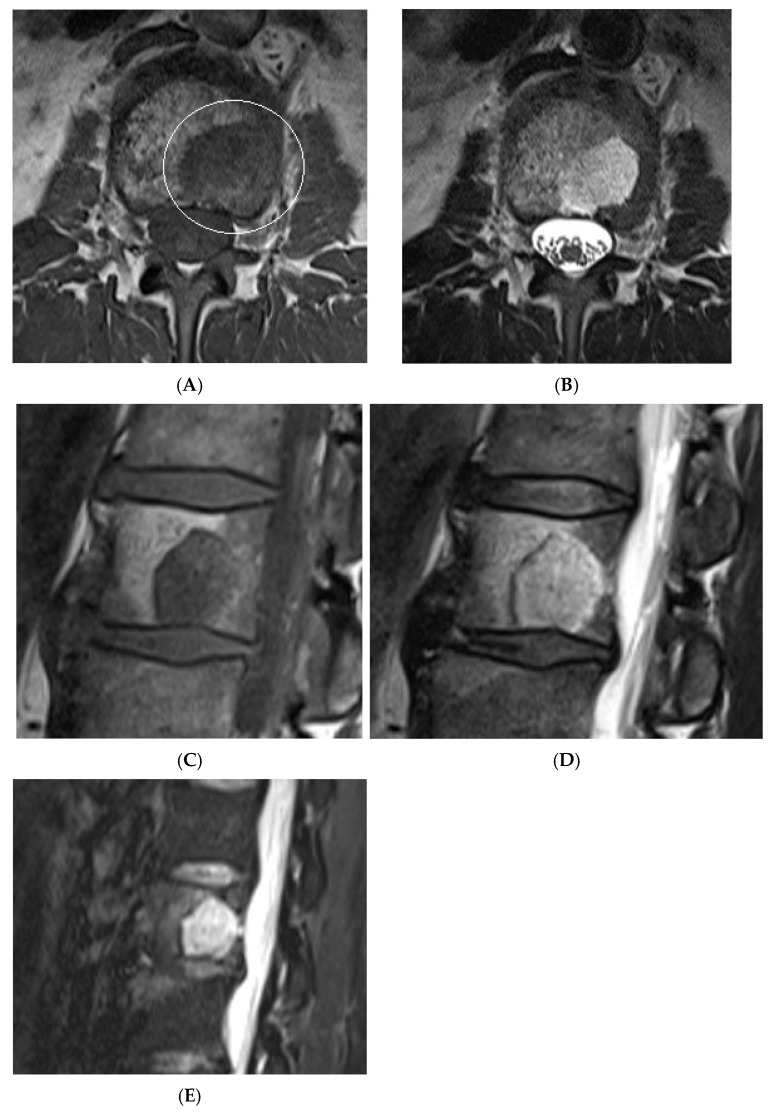
Collision Lesions: Typical and atypical haemangioma of vertebral body. (**A**) Axial T1W MRI demonstrating a classic haemangioma with a T1 hyperintense lesion occupying the vertebral body, with a smaller atypical T1 hypointense lesion (circled) adjacent, in keeping with an atypical haemangioma. (**B**) Axial T2W MRI with the same lesions—the atypical lesion to the left side of the patient (vertebral body) has a higher vascular component and therefore is more hyperintense than the adjacent and larger typical haemangioma. The larger lesion still remains hyperintense on T2, in keeping with predominant internal fat content. (**C**) Sagittal T1W spine MRI. (**D**) Sagittal T2W spine MRI. (**E**) Sagittal STIR spine MRI demonstrating fat suppression within the typical haemangioma with hyperintense signal within the atypical haemangioma as a collision lesion example.

**Figure 15:**
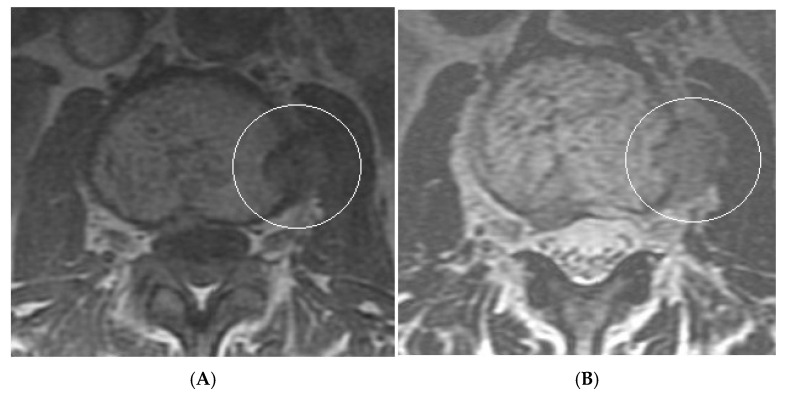
Collision Lesions: Renal cell carcinoma metastases within a haemangioma of vertebral body, Case 1. (**A**) Axial T1W MRI—A large T1 hyperintense lesion with typical appearances of a haemangioma within the vertebral body, associated with a smaller peripheral located T1 isointense lesion with extraosseous component (circled). (**B**) Axial T2W MRI—Same case with T2 hyperintense vertebral body lesion confirming internal fat content and the eccentrically located T2 hyper- to isointense lesion proven to be a renal cell carcinoma on histology.

**Figure 16:**
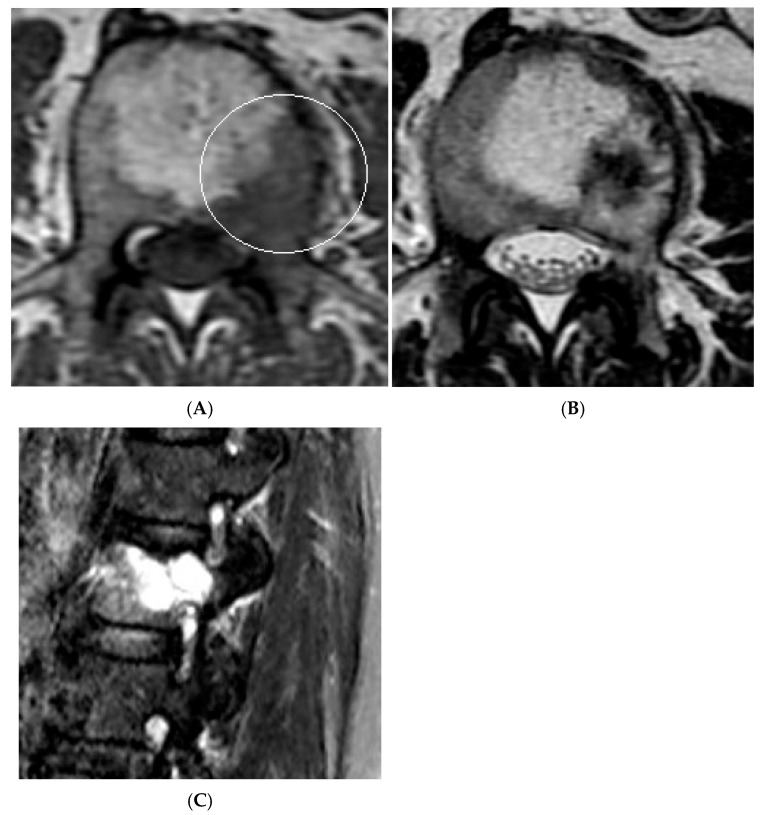
Collision Lesions—Renal cell carcinoma metastases within a haemangioma of the vertebral body, Case 2. (**A**) Axial T1W MRI with a typical vertebral body haemangioma with an abnormal low T1 signal in its posterolateral aspect with periosteal change (circled). (**B**) Axial T2W MRI demonstrates the aggressive lesion within and adjacent to the typical haemangioma in image (**A**). (**C**) Sagittal STIR MRI—Fat-suppressed haemangioma with a hyperintense (biopsy-proven) metastases in its posterior aspect involving the pedicle.

Collision lesions of the spine are rare, with few documented cases involving the spinal cord. Notably, metastases from renal cell carcinoma to spinal hemangioblastomas in patients with Von Hippel–Lindau syndrome have been reported [21]. However, collision lesions specifically involving vertebral haemangiomas are exceedingly uncommon, with only a few cases of metastatic infiltration into haemangiomas described in the literature. These include metastases from renal cell carcinoma [22,23], colonic adenocarcinoma [24], and lymphoma [25], each leading to varying clinical manifestations, including spinal cord compression [25] (Figure 14, Figure 15 and Figure 16).

In a comprehensive case series, collision lesions with both typical and atypical haemangioma components as well as focal marrow hyperplasia were described [23]. A potential explanation for the latter phenomenon may lie in the histological subtype of vertebral haemangiomas; i.e., the capillary type contains intervening normal bone tissue, which may support hematopoietic proliferation, unlike the cavernous variant. This suggests that in rare cases, capillary-type osseous haemangiomas may serve as a substrate for focal marrow hyperplasia [23].

## 3. Differentiating Benign from Malignant Lesions Using Macroscopic and Microscopic Fat

### 3.1. Macroscopic Fat

Macroscopic fat is visible on standard imaging techniques:

- Radiographs: Fat appears as radiolucent areas.
- CT: Fat has a Hounsfield unit (HU) typically between −100 and −50, with an optimal range defined as −140 to −30 HU [26].
- MRI: Fat is hyperintense to muscle on T1-weighted sequences and suppressed on fat-saturated sequences.

Macroscopic fat is uncommon in malignant lesions, making its presence a reassuring sign. Exceptions for this include liposarcomas [27]. While soft tissue liposarcomas are relatively common, primary intraosseous liposarcoma remains an exceedingly rare entity. More frequently, malignancy arises through the transformation of a pre-existing fat-containing bone lesion. A definitive diagnosis requires the histopathological confirmation of liposarcomatous tissue without evidence of other tumour components. Radiologically, the presence of a cortical breach within an otherwise benign-appearing fatty lesion should raise suspicion and warrant further evaluation with multiparametric MRI, contrast-enhanced MRI, or biopsy. Areas of atypical enhancement, architectural distortion, or restricted diffusion may necessitate targeted biopsy to exclude malignant transformation [27].

The literature search also revealed a case of post-treatment follow-up of breast cancer lytic bone metastases demonstrating substantial fatty replacement within the lesions, as evidenced by a marked shift in HU values from an initial 109 HU to 122 HU to a post-systemic treatment value of −10 HU. Consequently, in larger bone lesions where HU measurement is reliable, assessing for fat replacement on follow-up imaging may serve as a useful indicator of therapeutic response [28].

### 3.2. Microscopic Fat

Microscopic fat may not be visualised directly, but its presence indicates preserved marrow fat, which favours benign processes.

MRI remains the modality of choice for detecting and characterising bone marrow lesions, particularly metastatic disease. T1-weighted and fat-suppressed fluid-sensitive sequences are routinely employed for this purpose. Fat suppression in MRI can be achieved through several techniques: short tau inversion recovery (STIR), chemical shift selective saturation (CHESS), and chemical shift-based water-fat separation methods in CSI, also known as Dixon technique [29].

Unlike conventional fat suppression techniques, the Dixon method achieves this in the post-processing rather than at the time of image acquisition, offering more consistent fat suppression across various field strengths and anatomical regions. It is compatible with spin echo, fast spin echo, and spoiled gradient-recalled echo sequences, and can be applied to T1-weighted, T2-weighted, and proton density sequences, including post-contrast acquisitions [29].

In spinal imaging, the Dixon method improves fat suppression uniformity, reduces susceptibility artefacts compared to other fat suppression techniques, and delivers a higher signal-to-noise ratio with reduced acquisition time. These advantages make it particularly effective for assessing marrow lesions and vertebral pathologies. The usefulness of CSI has therefore been quoted, reported, and described in many studies [30].

Given that malignant lesions usually replace normal marrow and lack microscopic fat, they produce minimal or no signal drop on CSI due to the paucity of fat compared to normal bone marrow [31].

## 4. Chemical Shift Imaging (CSI)

### 4.1. Principles of CSI

Chemical shift imaging (CSI), also referred to as Dixon imaging [31], is a specialised MRI technique that exploits the natural differences in precession frequency between fat and water protons. Since its introduction, CSI has become an indispensable tool in musculoskeletal radiology, particularly for evaluating bone marrow lesions. It is especially valuable for distinguishing benign fat marrow from malignant marrow-infiltrating processes, without the need for contrast agents or invasive procedures [32,33,34,35].

At the core of CSI lies the difference in the resonant frequencies of hydrogen nuclei in fat and water molecules. This difference is known as the chemical shift. Chemical shift imaging exploits this slight difference in resonance frequencies between fat and water protons, which causes their signals to periodically align (in-phase) and oppose (out-of-phase). At 1.5 T, this cycle occurs every 2.2 ms (after 1.15 ms at 3 T), resulting in in-phase signals at 4.4 ms and out-of-phase at 2.2 ms [31]. Gradient-echo sequences are particularly sensitive to this effect, as they do not employ a 180° refocusing pulse, allowing fat and water signals to add constructively or destructively depending on the echo time (TE) [29].

The Dixon technique, introduced in 1984 [36], builds upon this principle by acquiring images at two specific TEs, i.e., when fat and water are in-phase (IP) and out-of-phase (OP), thus enabling the separation of fat and water signals via image summation and subtraction. This results in four key image sets: in-phase, opposed-phase, fat-only, and water-only [37].

Clinically, Dixon imaging provides improved fat suppression, a wider field of view, reduced susceptibility artefacts, and enhanced signal-to-noise ratio, features especially beneficial in spinal MRI for evaluating marrow lesions and bony pathologies. Furthermore, the Dixon technique allows quantitative fat-water fraction analysis within lesions, supporting its role in differentiating benign from malignant marrow conditions and in assessing treatment response.

### 4.2. Physics of Chemical Shift Imaging

Fat and water protons occupy distinct chemical environments, leading them to experience slightly different local magnetic fields within the scanner. Fat molecules, being larger, contain multiple carbon and hydrogen atoms in chains of varying length, are surrounded by more free electrons, which generate a microscopic opposing field, a phenomenon known as “magnetic shielding”. This secondary magnetic field reduces the effective magnetic field experienced by fat protons compared to water protons, causing fat to precess at a slightly lower frequency.

At 1.5 Tesla, this difference is approximately 210 Hz (3.5 ppm), primarily due to the methylene component of fat, which dominates the fat spectrum. Although the fat signal comprises multiple peaks, the principal resonance is sufficient for most clinical applications. This frequency offset results in periodic phase differences between fat and water, forming the basis for both chemical shift artefacts and fat–water separation techniques [37]. By comparing signal intensity (SI) values in the same voxel on both in-phase and out-of-phase images, one can quantify the proportion of fat and water in the tissue. This is done by reviewing the lesion of concern on the in- and out-of-phase images, and drawing a region of interest (ROI) within the lesion corresponding to its centre. The mean SI values of the ROI computed by a PACS software are then used to calculate the percentage signal dropout from the following formula:

% SI drop = (SI^OP^/SI^IP^) × 100

The calculated percentage value is subtracted from 100 to give the exact numerical value of the actual signal drop-off. Therefore, bone lesions which show >20% SI drop on the OP vs. IP images are classed as fat-containing whilst those exhibiting <20% signal drop-off are considered fat-replacing or neoplastic. Studies have used this cut-off for 1.5 T scanners with a threshold of >25% for fat-containing and <25% for fat-replacing lesions, respectively, for 3 T scanners [38].

### 4.3. Diagnostic Criteria

**Figure 17:**
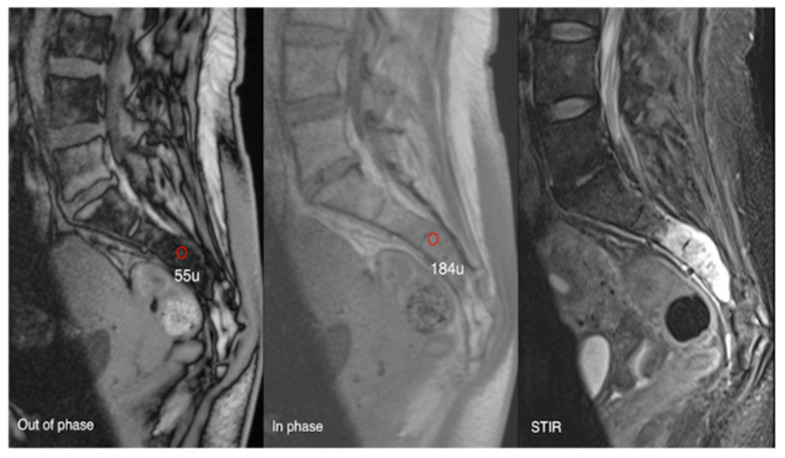
Aggressive sacral haemangioma with soft tissue mass. Out-of-phase, in-phase, and STIR sagittal MRI spine as labelled on images (**bottom left**), showcasing the expansile nature of the lesion but also the internal fat content as demonstrated by >20% signal dropout of signal intensity values (55 SI vs. 184 SI) on the out-of-phase vs. in-phase sequence.

**Figure 18:**
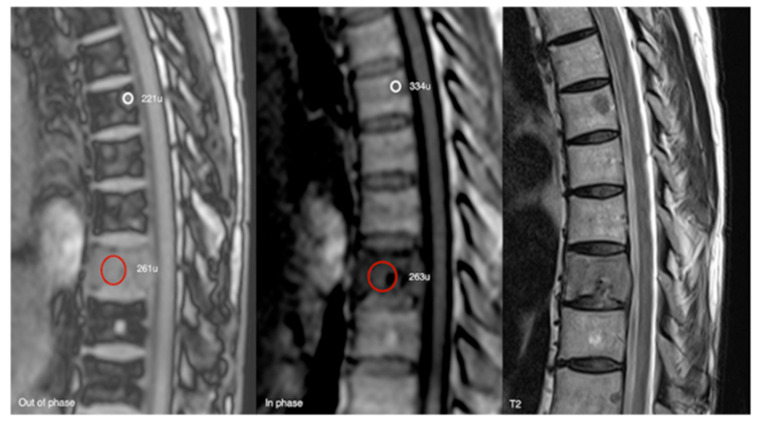
Out-of-phase, in-phase, and T2 sagittal MRI spine as labelled on images (**bottom left**). Typical haemangioma, upper thoracic spine (221 SI vs. 334 SI), and biopsy-proven breast metastases, lower thoracic region (261 SI vs. 263 SI), with respective values on out-of-phase and in-phase imaging. The calculated signal dropout in the case of the typical haemangioma is 33.8 (>20%) and for breast metastasis it is 0.8 (<20%).

**Figure 19:**
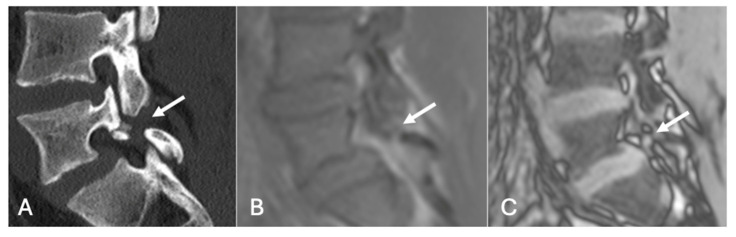
Pars defect assessment CT image (**A**) and chemical shift in-phase (**B**) and out-of-phase (**C**), showing pars defect of L5 (arrow).

**Figure 20:**
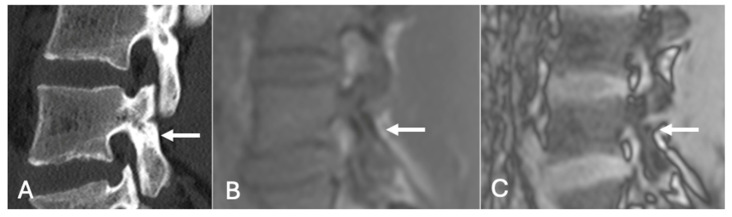
Pars defect assessment sagittal CT image (**A**) and chemical shift in-phase (**B**) and out-of-phase (**C**), showing intact pars of L4 (arrow).

Using a 20% signal drop-off threshold on chemical shift imaging (CSI), Davies et al. [39] observed a sensitivity of 91.7% and a specificity of 72.7% for distinguishing benign from malignant bone lesions. This suggests that while lesions with ≤20% signal loss should be considered indeterminate due to limited specificity, CSI can confidently exclude malignancy when signal drop exceeds 20%, given its high negative predictive value (97.1%). Accordingly, biopsy may be avoidable in lesions with >20% signal drop, whereas histological confirmation or follow-up remains advisable for indeterminate cases (Figure 17, Figure 18, Figure 19 and Figure 20).

### 4.4. Applications

**Figure 21:**
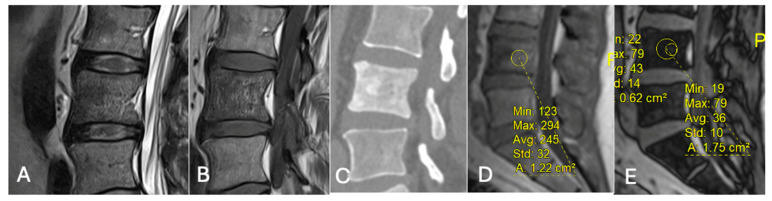
Focal marrow hyperplasia in L4. Sagittal T1 (**A**), T2 (**B**), CT (**C**), and chemical shift (**D**,**E**), showing heterogeneous signal on T1 and T2 and sclerosis on CT with significant signal drop on chemical shift images (**D**,**E**).

1. Differentiating Red Marrow Reconversion from Metastasis: Red marrow reconversion may mimic metastatic infiltration on T1-weighted images but retains microscopic fat and hence shows significant signal drop on CSI, unlike true metastases [40] (Figure 21).
2. Characterising Indeterminate Marrow Lesions: In patients with cancer, incidental marrow lesions often cause clinical concern. CSI helps identify those lesions with retained fat, reducing the need for biopsy or further imaging such as bone scan or positron emission tomography (PET) [39] (Figure 18).
3. On a slightly different theme, whilst not strictly utilising the SI drop-off calculations, chemical shift imaging in itself has been demonstrated to assess for and evaluate pars defects. In a cohort of 70 patients, chemical shift MRI effectively identified both intact and defective pars interarticularies, with out-of-phase imaging slightly better for detecting defects and in-phase imaging better for confirming intact pars. Although differences were not statistically significant, the technique demonstrated strong intra- and interobserver reliability and offers a fast, reliable complement to conventional MRI [41] (Figure 19 and Figure 20).

### 4.5. Limitations and Artefacts

While CSI is highly useful, it is not infallible. Fat–water swap artefacts can occur where there is a swap of fat and water-only voxels. This can affect up to 10% of routine Dixon imaging [42]. These can be confirmed as artefacts by reviewing the in-phase images [43].

The treatment of bone marrow lesions can also result in the development of intralesional fat, potentially leading to misinterpretation as benign, a diagnostic pitfall that also affects T1-weighted sequences. In such cases, clinical history and context are essential to prevent misdiagnosis [43].

A further discrepancy in signal dropout measurements between longitudinal (i.e., coronal/sagittal) and axial planes in 9–14% of skeletal marrow lesions was reported [38]. While the cause of this variation remains unclear, it is considered a true finding rather than an artefact, and did not significantly impact diagnostic accuracy, sensitivity, or specificity in the study [38]. Notably, longitudinal measurements more frequently correlated radiological with histopathological diagnoses, suggesting that they may be more reliable when discrepancies arise. This phenomenon highlights the importance of not relying solely on signal intensity drop on opposed-phase images for lesion classification, and reinforces the continued value of radiographs, especially in evaluating the growth rate of lytic lesions and matrix characteristics [38].

## 5. Conclusions

Bone lesions can pose diagnostic dilemmas, particularly in patients with known or suspected malignancy, where the stakes of misinterpretation are high, and therefore fat detection in bone lesions, either macroscopic or microscopic, offers valuable diagnostic information, particularly when reviewed with patient history and clinical presentation, as summarised in Table 1 and Table 2. Recognising typical features of benign fat-containing lesions, understanding the available imaging arsenal and specialised techniques such as CSI in particular, as a non-invasive aid, and maintaining awareness of atypical bone lesions and collision lesions can significantly improve diagnostic accuracy.

## Figures and Tables

**Table 1:** Practical imaging tips for lesion characterisation.

Clinical Context	Imaging Features Suggesting Benignity	Features Suggesting Malignancy
Age; Clinical history; Symptoms.	Well-defined borders; Central or peripheral fat (signal dropout on CSI); Absence of cortical breakthrough; No adjacent soft tissue mass.	Cortical destruction; Ill-defined margins; Soft tissue extension; Lack of microscopic fat (no signal dropout on CSI).

**Table 2:** When fat is reassuring vs. when it warrants further work-up.

Fat Is Generally Considered Reassuring When	Further Work-Up Is Needed When
Found in isolation without aggressive features; Lesion is stable over time; Consistent with classic benign lesion imaging features.	Fat is seen with concerning features (oedema, cortical breach) [27]; Lesion is enlarging or symptomatic; Patient has a history of cancer.
The careful interpretation of fat in context with lesion morphology and patient history is key to appropriate decision-making [38].	

## Abbreviations

Ppm	Parts per million
Hz	Hertz
Ms	Millisecond
